# Supplementary material for: Prevention of SIVmac251 reservoir seeding in rhesus monkeys by early antiretroviral therapy
Source: Nat Commun. 2018 Dec 21;9:5429. doi: 10.1038/s41467-018-07881-9 (PMC6303321; doi:10.1038/s41467-018-07881-9)
Supplement: Supplementary file 2 — Supplementary Information [file 41467_2018_7881_MOESM2_ESM.pdf]

Supplementary Methods

Viral sequencing. Virus sequencing of SIV recovered from plasma was performed essentially as described (*1*). Viral cDNA was diluted in 96-well plates to yield fewer than 30% wells positive for amplification to ensure that positive amplifications were a result of a single cDNA. Amplicons from cDNA dilutions resulting in less than 30% positive wells were sequenced. Raw cDNA sequence data was assembled using GeneCodes Sequencher DNA sequencing software. All assembled sequence contigs were manually corrected for individual ambiguous nucleotide errors and further quality controlled to exclude any amplicons derived from multiple templates. Nucleotide alignments were made using the GeneCutter algorithm as described below (http://www.hiv.lanl.gov/content/sequence/GENE_CUTTER/cutter.html).

Viral highlighter analysis. The *env* codon sequence alignments were manually reviewed after automatic alignment with Gene Cutter (hiv.lanl.gov/content/sequence/GENE_CUTTER/cutter.html) and iterated using highlighter tools.

Viral dynamics modeling. We examined the dynamics of reservoir seeding and rebound using relationships predicted from viral dynamics models (*2*). If each newly infected cell has some fixed probability of entering into latency, then the size of the latent reservoir should be proportional to the cumulative number of infections occurring before ART initiation [3]. Area-under-the-curve (AUC) viral load was used as a measure of the total amount of infections occurring, which is a reasonable approximation for acute SIV infection since $CD4^+$ T cell levels do not change consistently or significantly.

To calculate AUC viral load before ART in animals with detectable values, we fit the kinetics in each animal to a simple model of early exponential viral growth: $v(t) = v_0 e^{-at}$. Here v_0 is the effective infectious viral load from which the infection starts ($v(0)$), and a is the growth rate of viral load during acute infection. Values of viral load below the detection limit (50 copies/mL) were treated as censored data, and fitting was done using a maximum likelihood approach that assumes that the observed viral load is log-normally distributed around the true viral load, and that measurements include an error with variance σ^2. AUC was then as was calculated as:

$$AUC = \int_0^{t_{ART}} v(t)dt = v_0/a\,(e^{at} - 1)$$

To estimate AUC for animals treated after 3 or fewer days for which no detectable viral load was observed, we used the average values of $\log_{10}(v_0)$ and a from the remaining animals and the relevant t_{ART} value (6 hours, or 1, 2, or 3 days).

We then examined the relationship between pre-ART AUC viral load and measures of the long-lived reservoir. Because HIV/SIV DNA levels decay rapidly upon initial ART initiation and are assumed to be dominated at that time by labile forms, we used SIV DNA levels in the PBMC, LNMC, and GMMC after 6 months of ART (immediately prior to ART cessation). We implemented a regression method that accounted both for errors in both variables (AUC and SIV DNA) and censored data (SIV DNA values below the detection limit of 3 copies/10^6 cells). Log values of both variables were fit to a linear relationship to estimate the scaling coefficient between the variables. Spearman rank-order correlations were also performed to test the strength of the relationship. The fitted relationship was used to predict the exact values of undetectable SIV DNA values.

The probability of rebound ever occurring upon ART cessation, as opposed to "cure", is predicted by viral dynamics models (*3*) to follow the simple relationship:

$$P_{rebound} = 1 - e^{-\frac{a\,f\,N_{LR}}{\delta}}$$

Where N_{LR} is the total body number of latently infected cells, a is the rate (per day) at which any individual cell reactivates from latency ($1/a$ is the average time until reactivation), f is the probability that any individual reactivated cell will successfully establish infection, and depends on both the mean and variance of secondary infections and potentially on the probability of immune escape, and δ is the death rate (per day) of latently infected cells ($\ln 2\,/\delta$ is the half life of cells in the reservoir).

We examined the ability of different measures of the reservoir to predict the probability of rebound using a relationship inspired by this equation:

$$P(rebound) = 1 - e^{-\alpha\, m_{LR}}$$

Where we assume that the parameters a, δ, and f are the same for all animals and can be captured, along with the scaling between total reservoir size N_{LR} and reservoir metric m_{LR}, with the constant α. Fitting was done using a maximum likelihood approach and the following reservoir metrics: pre-ART AUC VL (observed or inferred), and pre-interruption SIV DNA in PBMC, LNMC, and GMMC (actual value if above the detection limit and inferred value based on previously fitted relationship to pre-ART AUC VL).

Supplementary Figures

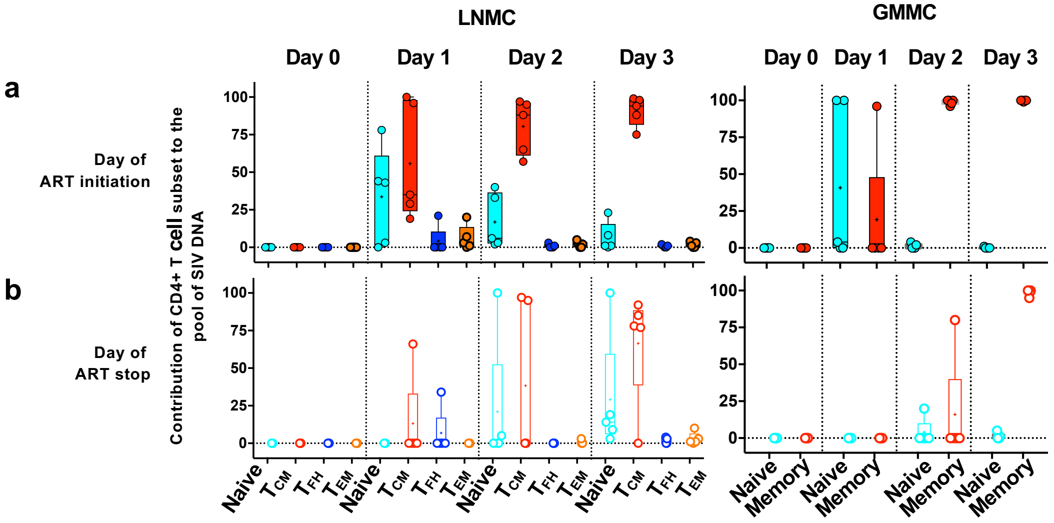

Supplementary Figure 1. Contribution of CD4+ T cell subsets to the pool of SIV DNA from infected, ART-treated animals. (a) Shown is the CD4+ T cell subset contribution to the SIV DNA reservoir of at the time of ART initiation. (b) Shown is the frequency of SIV DNA in each subset from LNMC or GMMC after 24 weeks of ART suppression. All data points are shown with minimum and maximum. The median value is represented by "+".

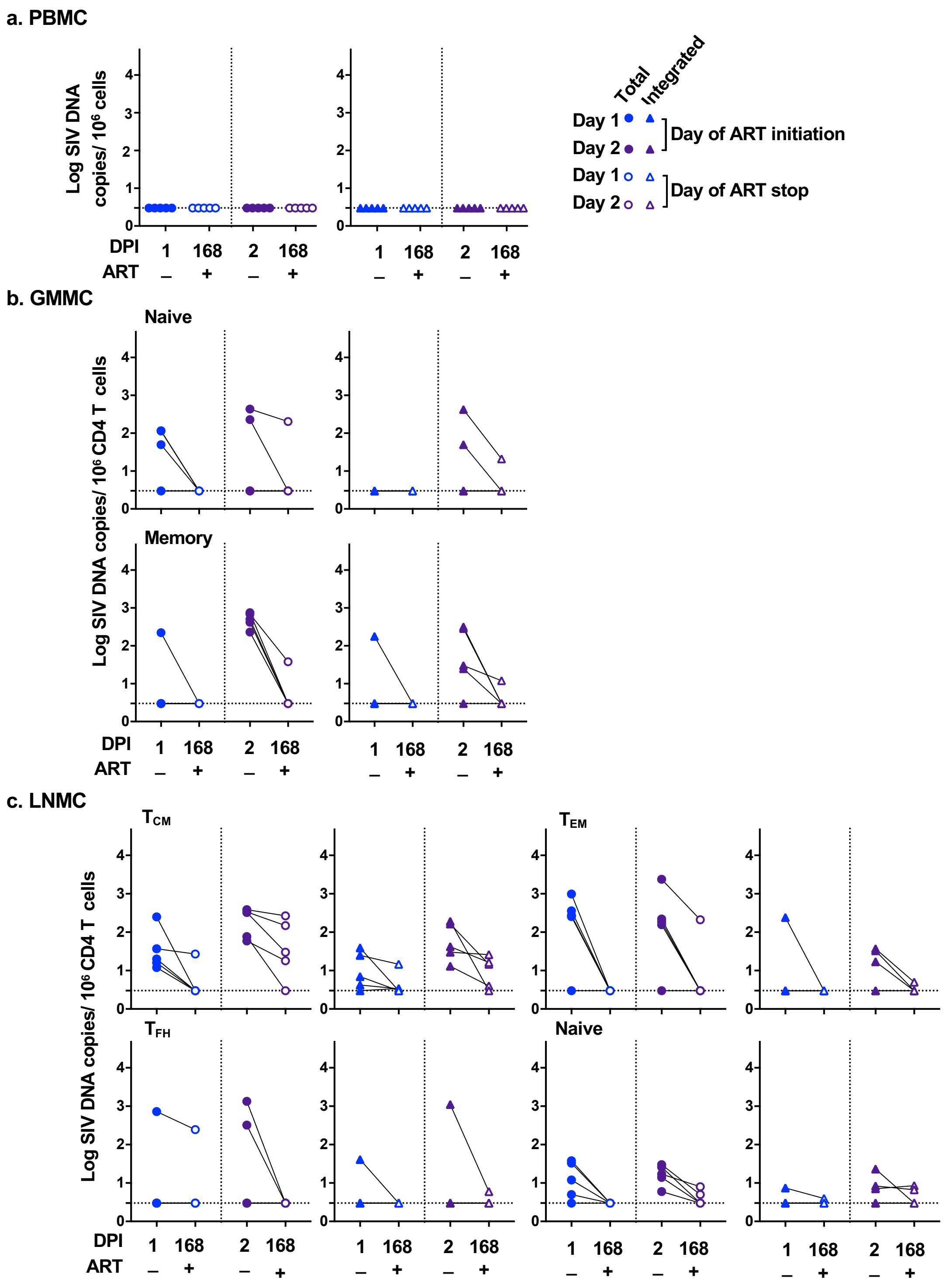

Supplementary Figure 2. Total versus integrated SIV DNA in the blood and tissues at pre-ART and ART cessation. Total and integrated SIV DNA in multiple tissues from two groups of animals that initiated ART on day 1 or 2 following SIV infection was quantitated prior to ART and at the time of ART discontinuation using a standard gag qPCR and a modified Alu-LTR PCR, respectively. Levels of total and integrated SIV DNA in PBMC (**a**), sorted naïve and memory CD4 T cells in GMMC (**b**), and naïve, central memory (T_{CM}), follicular helper (T_{FH}) and effector and effector memory (T_{EM}) CD4 T cells in LNMC (**c**) are shown. Assay sensitivity is indicated as not detected (ND, the hatched line) for both assays.

a

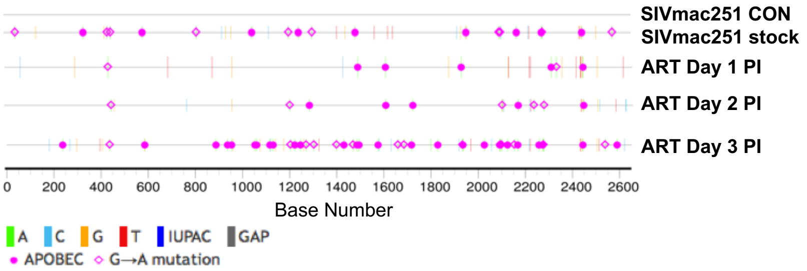

Supplementary figure 3a

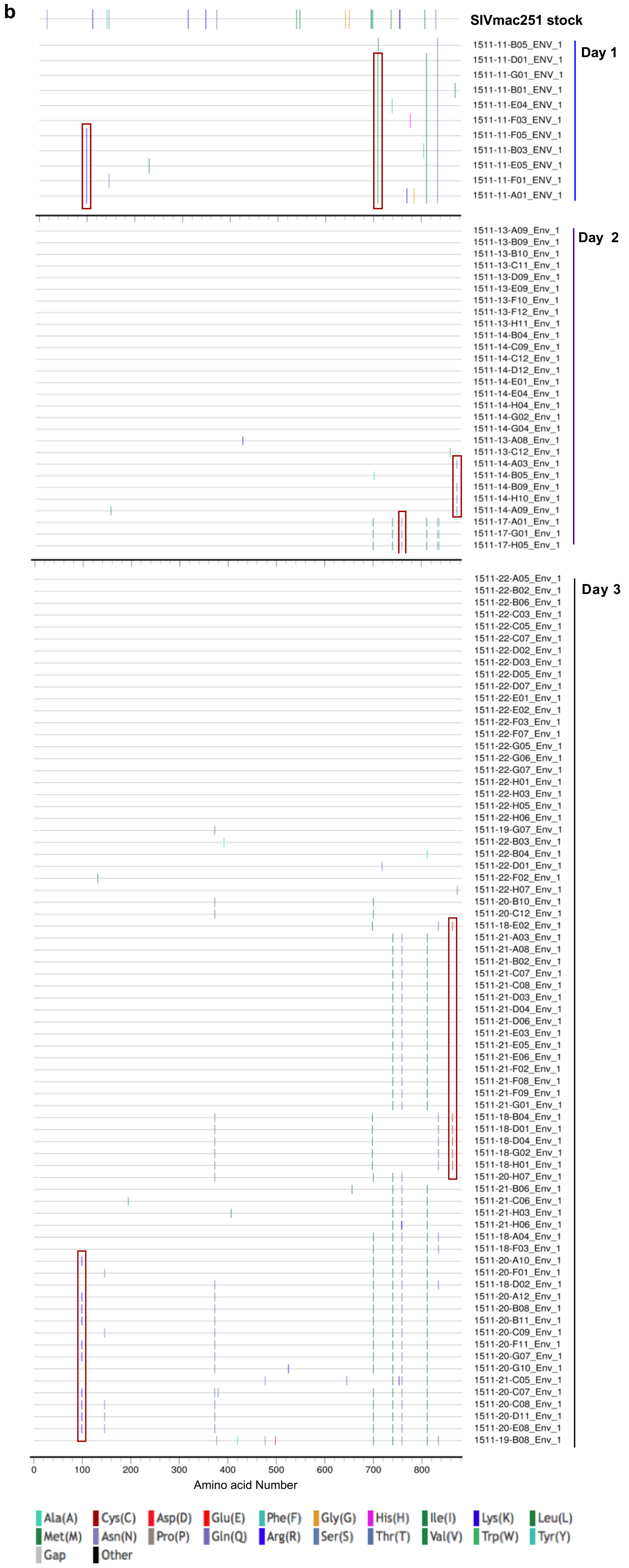

Supplementary figure 3b

Supplementary Figure 3. Highlighter alignment of *env* sequences from macaques after ART stop and viral rebound. Highlighter analyses of (**a**) nucleotide and (**b**) amino acid sequences are shown as compared to the inoculum sequences. A total of 112 sequences (day 1: 11, day 2: 28 and day 3: 73) were generated by SGA and compared with 37 sequences from the inoculum and to the consensus sequence of the SIVmac251 challenge stock. Nucleotide polymorphisms are indicated by a colored tic mark (adenine in green, cytosine in blue, guanine in orange and thymine in red). APOBEC-mediated G-to-A mutations are indicated by purple diamonds, and gaps (deletions) are indicated by gray tics (compressed). Amino acids that differ from the consensus sequence of the SIVmac251 challenge stock are indicated in color. Amino acid substitutions found in more than one sequences and only in plasma SIV isolated from macaques between 28 and 42 days post- ART stop are shown.

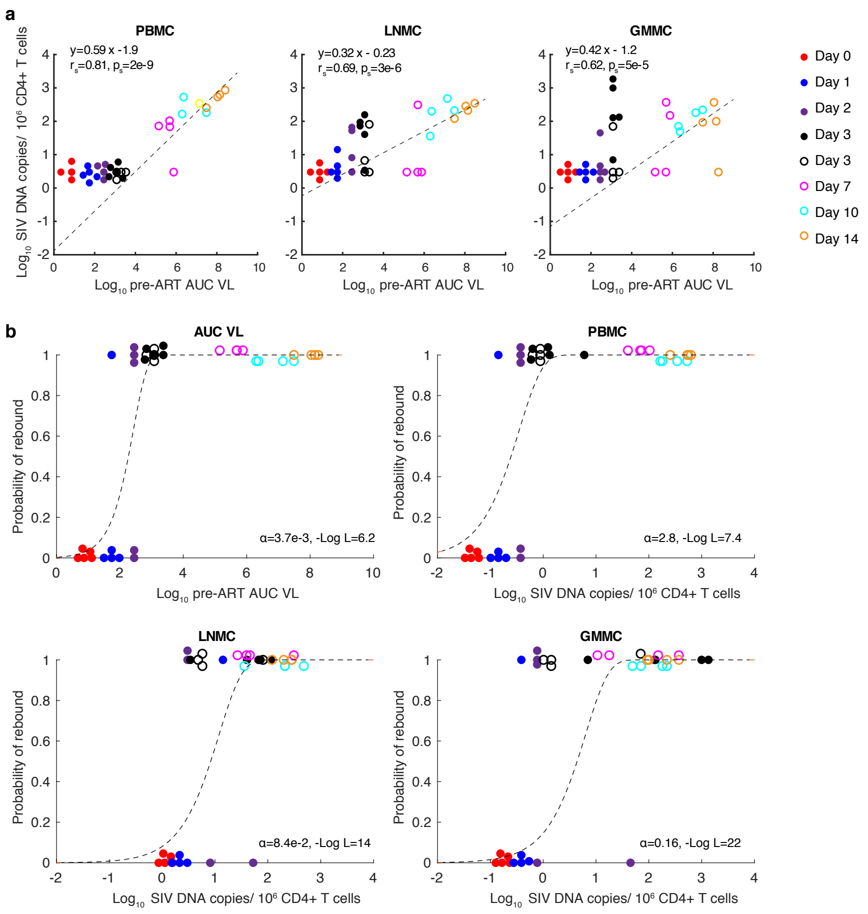

Supplementary Figure 4. Relationship between viral burden during acute infection, latent reservoir size, and the probability of remission following ART cessation. (a) Relationship between area-under-the-curve (AUC) of viral load before ART and SIV DNA measured at the time of ART cessation. Linear regression on $\log_{10}$ data was performed taking into account error in both variables and censored data (SIV DNA < 3 copies per 10^6 cells). Spearman rank-order correlations were also performed (r_s, p_s). **(b)** Relationship between measures of the reservoir size (SIV DNA in PBMC, LNMC or GMMC at ART cessation, or pre-ART AUC VL) and the probability of rebound (vs remission). Individual animals are plotted as 1 if they rebounded during the study period and 0 if they didn't. Data were fit to the model $P(rebound) = 1 - e^{-\alpha m_{LR}}$ where m_{LR} is one of the LR measurements. Negative log likelihood values are shown for the maximum likelihood α value; smaller values indicate better explanatory power of the model. AUC VL values for animals with no detectable viremia pre-ART were estimated based on the kinetics in other animals extrapolated backwards in time. SIV DNA values below the limit of detection were estimated based on AUC VL values for the same animals and the relationships shown in (**A**). Data point jitter is added to identical points for visualization purposes only. Details of fitting methods are found in the Supplemental Methods.

Supplementary References

1. J. B. Whitney *et al.*, Genital tract sequestration of SIV following acute infection. *PLoS Pathog* **7**, e1001293 (2011).
2. N. M. Archin *et al.*, Immediate antiviral therapy appears to restrict resting CD4+ cell HIV-1 infection without accelerating the decay of latent infection. *Proc Natl Acad Sci U S A* **109**, 9523-9528 (2012).
3. A. L. Hill, D. I. Rosenbloom, F. Fu, M. A. Nowak, R. F. Siliciano, Predicting the outcomes of treatment to eradicate the latent reservoir for HIV-1. *Proc Natl Acad Sci U S A* **111**, 13475-13480 (2014).
